# Combinative evaluation of primary tumor and lymph nodes to predict pelvic lymphatic metastasis in cervical cancer: an integrated PET-IVIM MRI study

**DOI:** 10.1186/s40644-020-00298-y

**Published:** 2020-03-06

**Authors:** Chen Xu, Xiaoran Li, Yanchi Shi, Bo Wang, Hongzan Sun

**Affiliations:** 1 Department of Radiology, Shengjing Hospital of China Medical University, Sanhao Street No36, Heping District, Shenyang, Liaoning PR China 110004; 2 Liaoning Provincial Key Laboratory of Medical Imaging, Sanhao Street No36, Heping District, Shenyang, 110004 Liaoning China

**Keywords:** Cervical cancer, Positron-emission tomography, Diffusion magnetic resonance imaging, Lymph node

## Abstract

**Background:**

The aim of this study was to evaluate the value of combining pelvic lymph node and tumor characteristics on positron emission tomography-intravoxel incoherent motion magnetic resonance (PET-IVIM MR) imaging for predicting lymph node metastasis in patients with cervical cancer, especially in those with negative lymph nodes on PET.

**Methods:**

The medical records of 95 patients with cervical cancer who underwent surgical resection with pelvic lymph node dissection were evaluated. The patients were divided into negative and positive groups according to postoperative pathologic lymph node diagnosis, and comparisons of the PET and IVIM-derived parameters between the two groups were performed. Univariate and multivariate analyses were performed to construct a predictive model of lymph node metastasis.

**Results:**

For all patients, tumor SUV_max_, TLG, D_min_, PET and MRI for lymph node diagnosis showed significant differences between patients with and without confirmed lymph node metastasis. Univariate and multivariate logistic analysis showed that the combination of tumor TLG, D_min_ and PET for lymph node diagnosis had the strongest predictive value (AUC 0.913, *p* < 0.001). For patients with PET-negative lymph nodes, SUV_max_, SUV_mean_, MTV, TLG, and D_min_ showed significant between-group differences, and univariate and multivariate logistic analysis showed that TLG had the strongest predictive value.

**Conclusions:**

The combination of tumorTLG, D_min_ and PET for lymph node diagnosis is a powerful prognostic factor for all patients. TLG has the best predictive performance in patients with PET negative lymph nodes.

## Background

Cervical cancer, one of the common malignant tumors of the female reproductive system, is a serious threat to women’s health and life [1]. Local recurrence and distant metastasis are the main causes of death. At present, treatment and prognosis plans for patients with cervical cancer are mainly based on the Federation International of Gynecology and Obstetrics (FIGO) stage. According to FIGO 2018 for cervical cancer, regardless of tumor size and parametrial infiltration, the involvement of lymph node metastasis is classified as stage IIIC. Therefore, accurate diagnosis of lymphatic metastasis is crucial for developing individualized treatment plans, improving prognosis, and reducing mortality [2–4].

Recently, fluoro-D-glucose (FDG)-positron emission tomography (PET) has been applied to the diagnosis of cervical cancer metastatic lymph nodes. Rather than computed tomography (CT) or magnetic resonance imaging (MRI), which identifies lymphatic metastasis according to short diameter length and morphology [5].FDG-PET provides quantified metabolic information about lymph nodes and is widely used in the evaluation of lymph node metastasis [6]. One study has proved the prognostic value of lymph node metabolism information in patients with cervical cancer [7]. A lymph node with significantly higher FDG metabolism than the background level is defined as a PET-positive lymph node [8, 9]. However, FDG is not a specific imaging agent, and lymph node reactive hyperplasia is also characterized by high metabolism resulting in many false positive cases. Also, the limited resolution of PET and partial volume effect will also affect the diagnostic accuracy of small lymph nodes.

Therefore, quantitative analysis of lymph nodes alone is ambiguous to achieve satisfied accuracy for predicting lymph node metastasis. In this study, we present a combined model (PET and intravoxel incoherent motion (IVIM)-derived imaging of primary tumors and PET/MRI diagnosis of lymphatic metastasis) for predicting lymphatic metastasis confirmed by postoperative pathology in all patients and patients with PET-negative lymph nodes.

## Methods

### Patients

We retrospectively collected the medical records of patients with a high suspicion of cervical cancer who had underwent PET-IVIM MRI in our hospital from April 2017 to September 2019. The study participants met the following inclusion criteria: (1) underwent surgery; (2) did not receive radiotherapy or chemotherapy before surgery; and (3) had confirmed cervical cancer via postoperative pathology.

### Pathologic diagnosis

Postoperatively, the tissue was transported to the pathology department of our hospital. Hematoxylin-eosin (HE) stained sections were evaluated by two pathologists with more than 10 years of experience. The following information was recorded: lymph node metastasis, histologic tumor type, cervical stromal invasion depth, and tumor differentiation grade. The patient with one or more pelvic metastatic lymph nodes identified with pathological results was regarded as a positive case.

### PET-MRI scanning and image acquisition

**Fig. 1:**
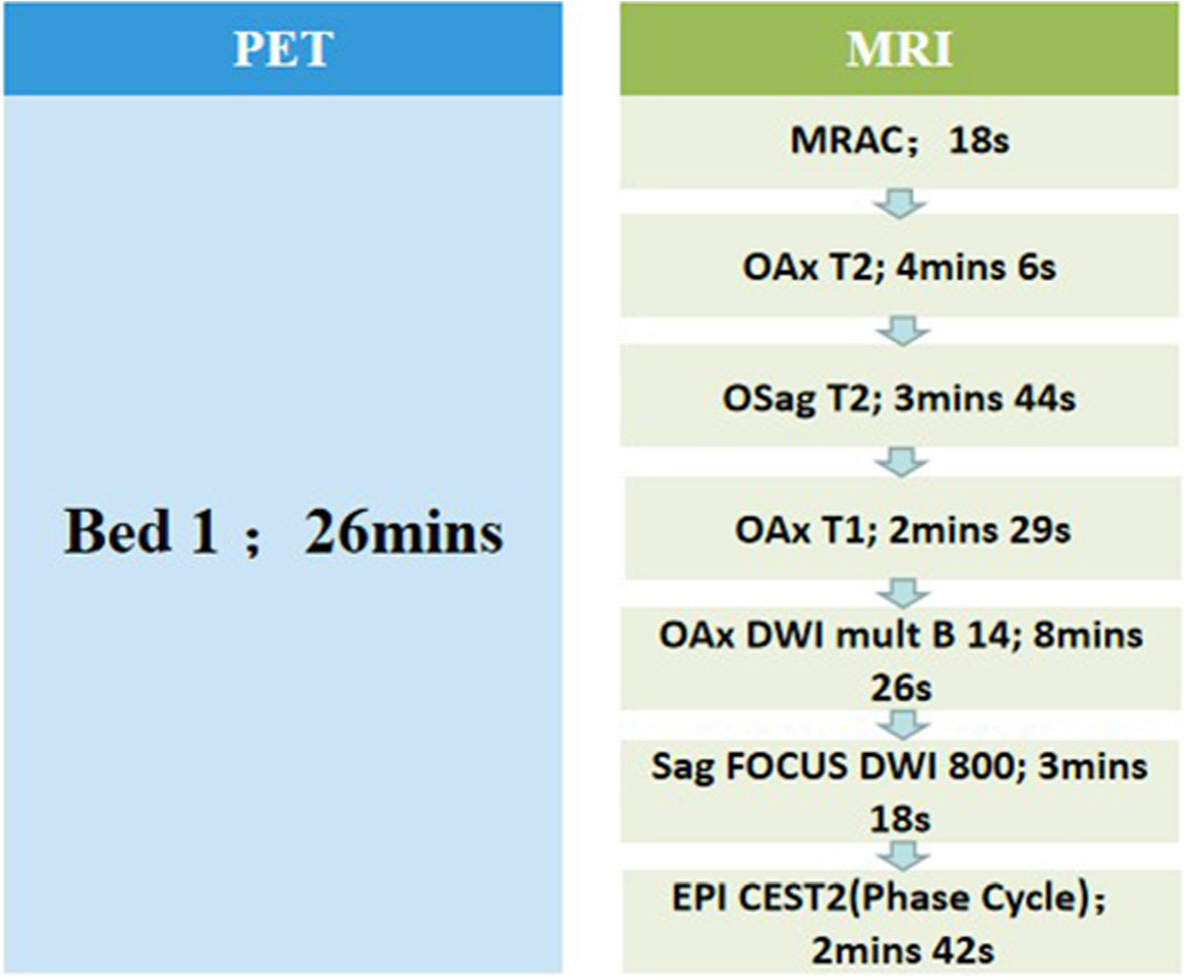
PET and MRI scan at the same time, each sequence of MRI is performed in sequence, the total time is about 26 min

All patients underwent GE Signa integrated PET/MRI (Signa, GE Healthcare), which combined a 3.0 T MRI scan (GE Signa 750w) and TOF-PET, allowing simultaneous collection of both PET and MRI data. Before the examination, the patients fasted for 4 to 6 h with measured blood glucose levels lower than 7.0 mmol/L. ^18^F-FDG (4 MBq/kg) was injected through the cubital vein in the resting state, and PET/MRI was performed after 60 ± 12 min with a 32-channel coil (Upper Anterior Array, UAA). For PET scanning execution, a Dixon MRI sequence was used to attenuate the gamma rays, and LIST-mode and the ordered subset maximum expected iteration method were used to reconstruct the images. The MR-IVIM imaging parameters are summarized in Table 1. PET/MRI acquisition sequence and durations are shown in Fig. 1.

**Table 1:** Parameters used for MR-IVIM imaging

	Axial T1	Axial T2	Sagittal T2	DWI	Axial IVIM
TR	500 ms	498 ms	4323 ms	4000 ms	6900 ms
TE	8 ms	79 ms	65 ms	238 ms	minimum
Thickness	6.0 mm	6.0 mm	6.0 mm	6.0 mm	8.0 mm
Interval	2.0 mm	2.0 mm	1.2 mm	2.0 mm	9.0 mm
FOV	26 cm	36 cm	24 cm	40 cm	40 cm
Matrix size	384 × 384	384 × 384	384 × 384	128 × 128	128 × 128
NEX	2	1.5	4	6	6
b-values (s/mm^2^)				0,800	0,10, 25, 50, 75,100,125,150, 200, 300, 400, 600, 800, and 1000

*TR* Repetition time, *TE* Echo time, *FOV* Field of view, *NEX* Number of excitations

### Image analysis

Image analysis using AW4.6 (GE Medical System) workstation was performed by 2 radiologists with more than 4 years of radiodiagnostic experience. After delineation of a lesion region of interest (ROI) at the optimal level of the PET image, the fused-PET/MRI software automatically calculated the metabolic tumor volume (MTV), total lesion glycolysis (TLG), and maximum and mean standardized uptake values (SUV_max_ and SUV_mean_) of the entire tumor. A 40% SUV_max_ threshold was used to calculate MTV [10, 11]. The IVIM data were analyzed using IMAgenGINE MRToolbox software (Vusion Tech Ltd). The IVIM formula was: Sb/S0 = F exp. [−b × (D* + D)] + (1-F) × exp-(b × D) [12]. On the axial T2-weighted image, the radiologists delineated ROIs at all levels of the lesion, and then the software automatically generated the volume of interest (VOIs) and copied them to the apparent diffusion coefficient (ADC) map, diffusion-related coefficient (D) map, perfusion related diffusion coefficient (D^*)^ map, and perfusion-related parameter (f) map, obtaining the parameters ADC_mean_, ADC_min_, D_mean_, D_min_, D^*^, and f.

### PET and MRI diagnosis of lymphatic metastasis

By comparing with surrounding background tissues, a lymph node with increased uptake by visual assessment is considered as a PET-positive lymph node, independently of node size. The method of PET image analysis was based on the previous literatures [8, 9]. Patients with one or more positive lymph nodes on PET imaging were considered to be patients with PET positive lymph nodes. Lymph nodes with short axis diameter greater than 1.0 cm and morphological characteristics on MRI were considered as MRI-positive lymph nodes [13, 14]. The results were independently evaluated by two radiologists with 12 and 13 years of experience in nuclear medicine and radiology.

### Statistical analysis

The data were analyzed using MedCalc software (version 15.2.2), and a *p* value less than 0.05 was considered as statistical significance. The patients were divided into negative and positive group according to postoperative pathological lymph node diagnosis. Differences in lymph node diagnosis by PET/MRI between groups were evaluated using the chi-square test. The values of PET- and IVIM-derived parameters of the primary tumor were used for the quantitative statistical analyses and the between-groups comparison was performed using the t-test or Mann-Whitney U test. Interclass correlation coefficient (ICC) was used to determine the consistency of the results recorded by the two radiologists (0.8–1 indicated high consistency) [15]. Prediction models for pelvic lymph node metastasis were constructed by univariate and multivariate logistic regression (variables with *p* < 0.1 in the univariate analysis were used in the multivariate analysis. Bonferroni corrections were performed to correct multivariate analysis). Receiver operating characteristic (ROC) curves were generated for combined or individual parameters to assess the area under the ROC curve (AUC) for differentiating the pelvic lymph node status and obtaining the cut-off threshold values corresponding to the highest value of the Jordan index. Comparison between the ROC curves was performed with the DeLong test. The correlation between parameters was analyzed by Spearman rank correlation.

## Results

### Patient characteristics

**Fig. 2:**
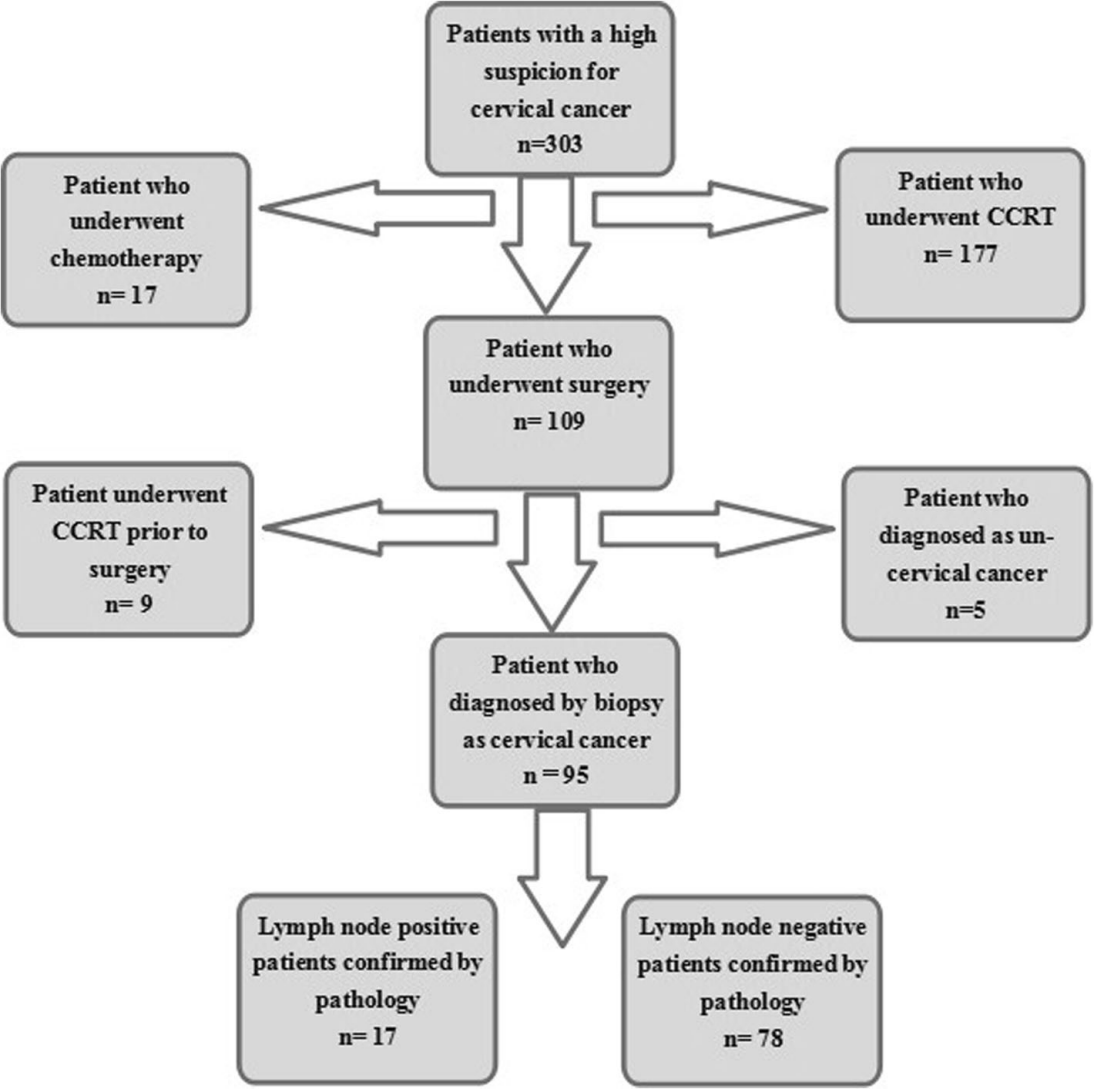
Flowchart of patients who were referred to the assessment

**Fig. 3:**
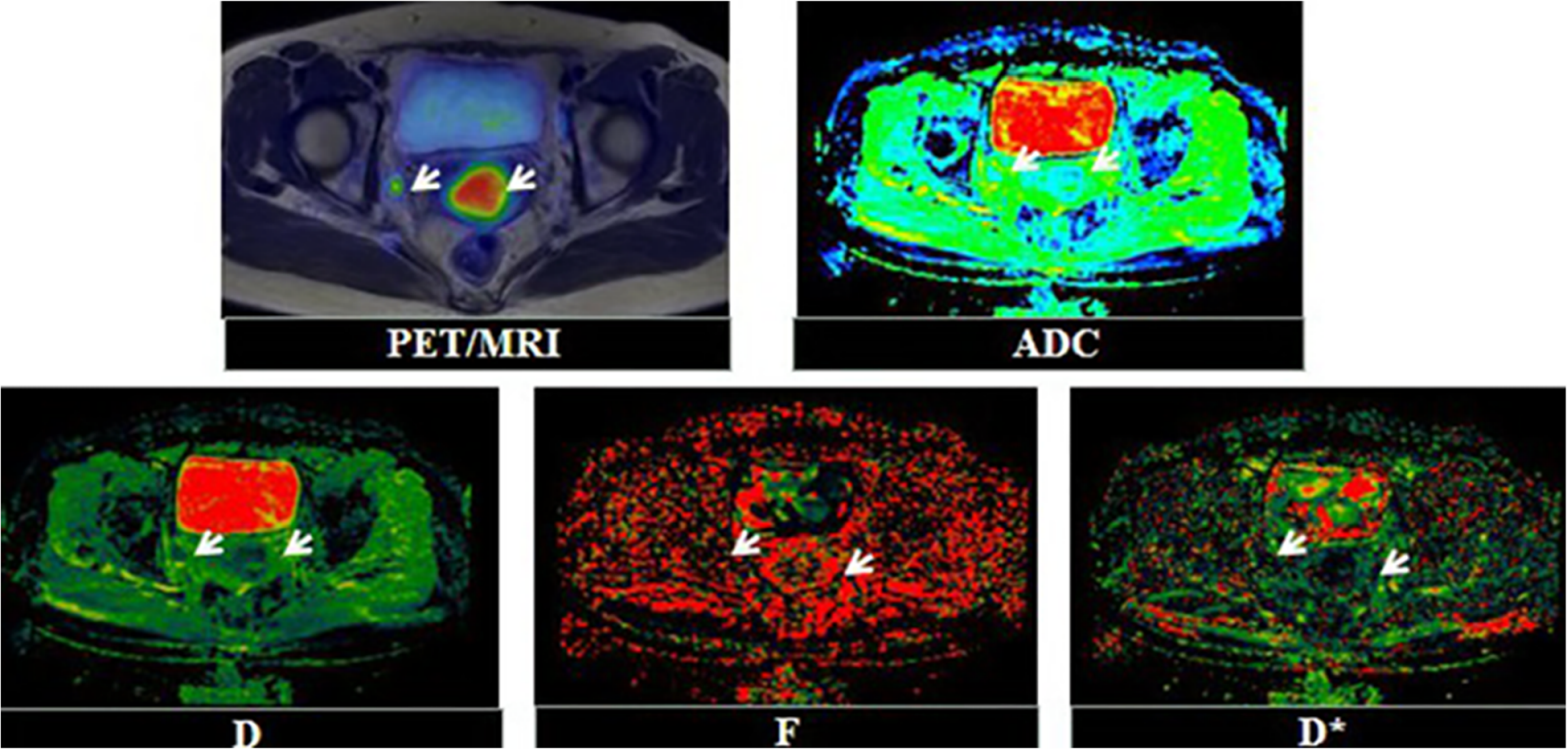
Forty-nine years-old female patient diagnosed with poorly differentiated squamous cervical carcinoma with a suspected lymph node metastasis showed on preoperative PET/MR. Postoperative pathology confirmed it as a metastatic lymph node. PET and PET/MR fusion images showed that FDG uptake in the tumor (SUV_max_ = 14.08 g/cm^3^, SUV_mean_ = 9.35 g/cm^3^, TLG = 326.69 g, MTV = 34.94 cm^3)^, and the ADC and IVIM image showed a high signal in the lymph node (ADC_mean_ = 1.27 × 10^− 3^ mm^2^/s, ADC_min_ = 0.67 × 10^− 3^ mm^2^/s, D_mean_ = 0.97 × 10^− 3^ mm^2^/s, D_min_ = 0.51 × 10^− 3^ mm^2^/s, D* = 35.92 × 10^− 3^ mm^2^/s, f = 0.17)

**Fig. 4:**
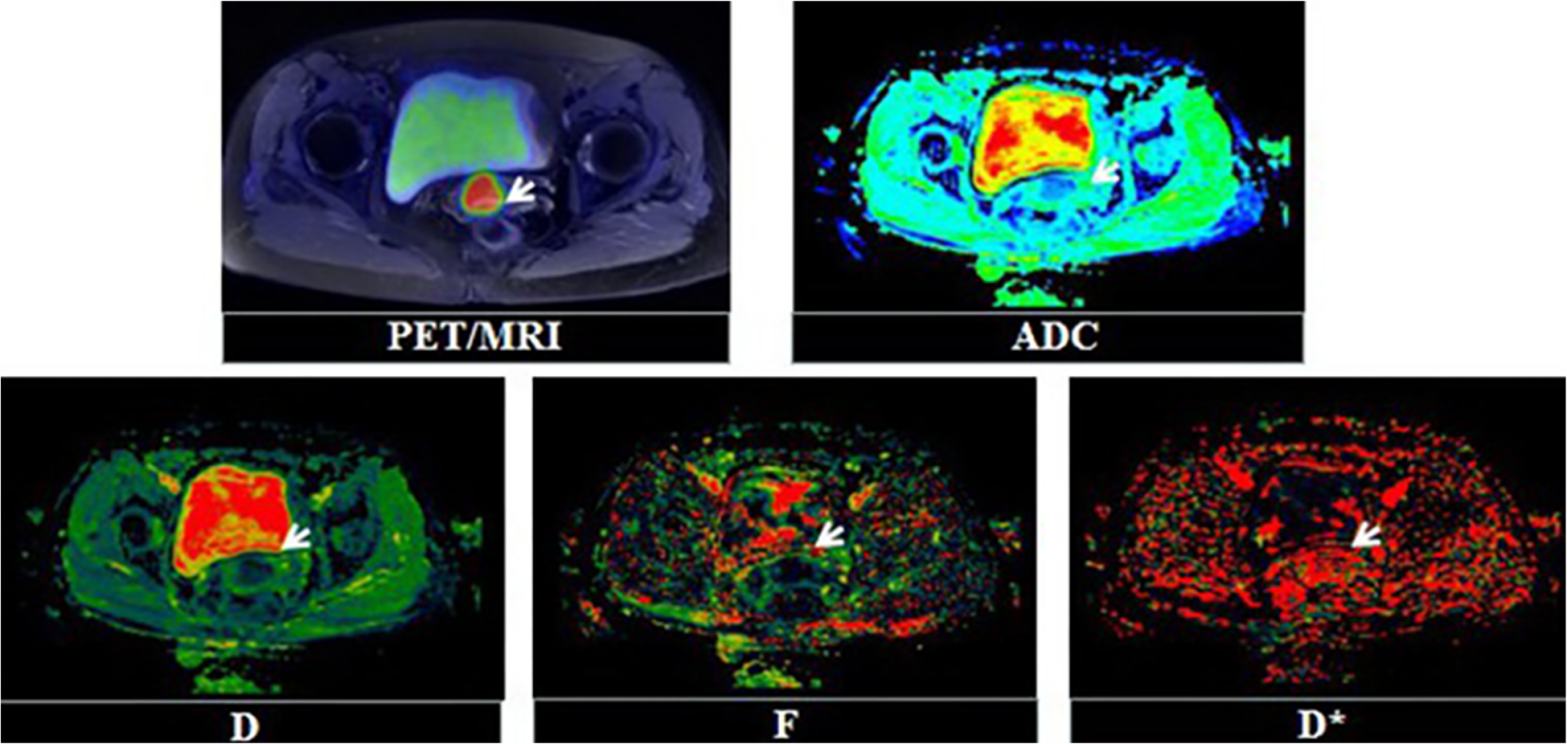
Fifty-nine years-old female patient diagnosed moderately differentiated, squamous cervical carcinoma, with no suspected lymph node metastasis showed on preoperative PET/MR, but it showed metastatic on postoperative pathology. The primary tumor has concentrated FDG uptakes on PET/MR fusion images (SUV_max_ = 11.68 g/cm^3^, SUV_mean_ = 7.92 g/cm^3^, TLG = 79.99 g, MTV = 10.1 cm^3^), and the ADC and IVIM image showed a high signal in the tumor (ADC_mean_ = 1.21 × 10^− 3^ mm^2^/s, ADC_min_ = 0.7 × 10^− 3^ mm^2^/s, D_mean_ = 0.87 × 10^− 3^ mm^2^/s, D_min_ = 0.5 × 10^− 3^ mm^2^/s, D* = 56.93 × 10^− 3^ mm^2^/s, f = 0.17)

Ninety-five patients were enrolled (Fig. 2): 78 without lymph node metastasis and 17 with lymph node metastasis (Figs. 3 & 4). The clinicopathological characteristics of the participants are summarized in Table 2.

**Table 2:** Patients’ clinicopathological characteristics

Clinical feature	Value
No. of patients	95
Mean age (range)	51.0 years (30–72)
FIGO stage (2018):	
Ib1	22 (23.2%)
Ib2	33 (34.7%)
Ib3	11 (11.6%)
IIa1	4 (4.2%)
IIa2	4 (4.2%)
IIb	4 (4.2%)
III	17 (17.9%)
Differentiation grade:	
Well differentiated	19 (20.0%)
Moderately differentiated	63 (66.3%)
Poorly differentiated	13 (13.7%)
Histologic type:	
Squamous carcinoma	89 (93.7%)
Adenocarcinoma	6 (6.3%)
Cervical stromal invasion depth:	
< ½	36 (37.9%)
≥ ½	59 (62.1%)
Pathological diagnosis of lymph nodes:	
Positive	17 (17.9%)
Negative	78 (82.1%)

FIGO stage (2018):Postoperative pathological staging

### ICC statistics

Interobserver agreements were excellent for tumor SUV_max_, SUV_mean_, MTV, TLG, ADC_mean_, ADC_min_, D_mean_, D_min_, D^*^, and f value (ICC,0.992, 0.991, 0.993, 0.993, 0.862, 0.822, 0.842, 0.811, 0.822 and 0.801, respectively).

### The relationship between lymph node metastasis and imaging parameters in all patients

In all patients, SUV_max_ (*p* = 0.048), TLG (*p* = 0.014), D_min_ (*p* = 0.020), MRI in lymph node diagnosis (*p* = 0.009) and PET in lymph node diagnosis (*p* < 0.001) showed significant differences between patients with and without lymph node metastasis (Table 3). The remaining PET- and IVIM-derived parameters did not show statistical differences between the two groups.

**Table 3:** The relationship between lymph node metastasis and imaging parameters in all patients

N	Negative	Positive	*P*
	78	17	
D_mean_ (10^− 3^ mm^2^/s)	0.90 ± 0.24	0.81 ± 0.16	0.131
D_min_ (10^− 3^ mm^2^/s)	0.59 ± 0.18	0.49 ± 0.13	0.020
ADC_mean_ (10^−3^ mm^2^/s)	1.13 ± 0.23	1.16 ± 0.26	0.988
ADC_min_ (10^−3^ mm^2^/s)	0.70 ± 0.14	0.76 ± 0.22	0.446
D* (10^−3^ mm^2^/s)	42.58 ± 15.35	42.87 ± 17.70	0.820
f	0.16 ± 0.06	0.18 ± 0.07	0.738
SUV_max_ (g/cm^3^)	15.35 ± 9.53	19.24 ± 8.69	0.048
SUV_mean_ (g/cm^3^)	9.16 ± 6.03	11.38 ± 5.32	0.061
MTV (cm^3^)	10.22 ± 8.06	16.67 ± 12.99	0.081
TLG (g)	100.11 ± 117.33	183.74 ± 144.08	0.014
PET in lymph node diagnosis			
PET negative lymph nodes	69	7	< 0.001
PET positive lymph nodes	9	10	
MRI in lymph node diagnosis			
MRI negative lymph nodes	61	8	0.009
MRI positive lymph nodes	17	9	

ROC analysis of the negative and positive groups according to postoperative lymph node diagnosis showed that SUV_max_ (AUC 0.654, *p* = 0.023), SUV_mean_ (AUC 0.646, *p* = 0.030), TLG (AUC 0.692, *p* = 0.009), D_min_ (AUC 0.681, *p* = 0.007), MRI in lymph node diagnosis (AUC 0.656, *p* = 0.020) and PET in lymph node diagnosis (AUC 0.736, *p* < 0.001) had a positive effect on predicting metastatic lymph nodes confirmed by postoperative pathology (Fig. 4). The optimal cut-off threshold values for SUV_max_, SUV_mean_, TLG, and D_min_ were 12.98 g/cm^3^ (sensitivity 76.47, specificity 53.85), 8.61 g/cm^3^ (sensitivity 70.59, specificity 60.26), 168.7 g (sensitivity 52.94, specificity 82.05), and 0.55 × 10^− 3^ mm^2^/s (sensitivity 82.35, specificity 56.41) respectively.

**Fig. 5:**
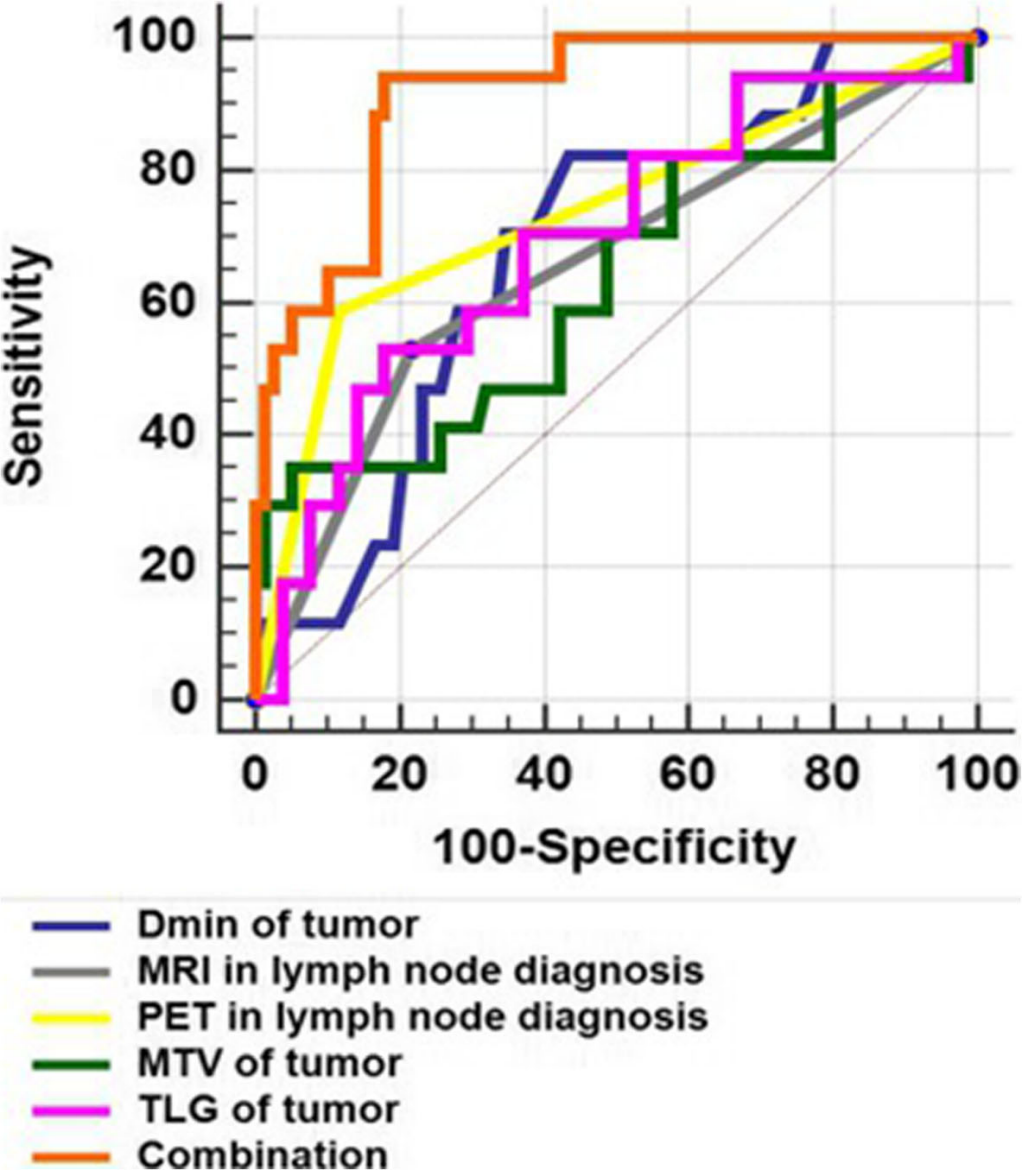
For all patients, ROC analysis shows that SUV_max_ (AUC 0.654, 95% confidence interval (CI) 0.549–0.749, *p* = 0.023), SUV_mean_ (AUC 0.646, 95% CI 0.541–0.741, *p* = 0.030), TLG (AUC 0.692, 95% CI 0.588–0.782, *p* = 0.009), D_min_ (AUC 0.681, 95% CI 0.577–0.773, *p* = 0.007), MRI in lymph node diagnosis (AUC 0.656, 95% CI 0.551–0.750, sensitivity 58.94, specificity 78.21, *p* = 0.020) and PET in lymph node diagnosis (AUC 0.736, 95% CI 0.636–0.822, sensitivity 58.82, specificity 88.46, *p* < 0.001) had a positive effect on predicting metastatic lymph nodes confirmed by postoperative pathology. The area under the ROC curve for the combination of TLG, D_min_ and PET/MRI in lymph node diagnosis (AUC 0.913, 95% CI 0.837–0.961, sensitivity 94.12, specificity 82.05, *p* < 0.001) was higher than any individual parameter (both, *p* < 0.05). CI: confidence interval

Univariate logistic analysis showed that MTV (*p* = 0.014), TLG (*p* = 0.019), D_min_ (*p* = 0.026), MRI for lymph node diagnosis (*p* = 0.012) and PET for lymph node diagnosis (*p* < 0.001) were associated with lymph node metastasis. Bonferroni corrected alpha value of 0.005 per test (0.05/10) was applied to the final multivariate analyses in exploring lymph node metastasis. The combination of TLG, D_min_, and PET for lymph node diagnosis had the strongest predictive value on multivariate logistic analysis (Table 4). The area under the ROC curve for the combination of TLG, D_min_, and PET for lymph node diagnosis (AUC 0.913, *p* < 0.001) was higher than that of any individual parameter (*p* < 0.05) (Fig. 5). The correlations between parameters are shown in Table 5.

**Table 4:** For all patients, univariate and multivariate analysis of parameters to predict lympha metastasis

Method: Stepwise; Dependent Y: Lymphatic metastasis Multivariate logistic regression: Enter Variable< 0.005				
Univariate logistic regression analysis				
Variable	coeffcients	Std Error	Wald	P
MTV	0.065374	0.026612	6.0348	0.014
TLG	0.0044791	0.0019118	5.4889	0.019
D_min_	−4.41339	1.98372	4.9498	0.026
PET in lymph node diagnosis	2.39356	0.60701	15.5488	< 0.001
MRI in lymph node diagnosis	1.39544	0.55797	6.2547	0.0124
Multivariate logistic regression analysis				
Variable	coeffcients	Std Error	Wald	P
TLG	0.0083138	0.0028803	8.3317	0.0039
D_min_	−10.14394	3.37033	9.0588	0.0026
PET in lymph node diagnosis	4.45475	1.10161	16.3528	0.0001

**Table 5:** Spearman rank correlation coefficients between parameters

	SUV_max_	SUV_mean_	TLG	MTV	ADC_mean_	ADC_min_	D_mean_	D_min_	f	D*
SUV_max_	…	0.980**	0.603**	0.126	− 0.039	− 0.039	− 0.079	− 0.257*	− 0.312*	−0.057
SUV_mean_	…	…	0.621**	0.135*	−0.027	−0.056	− 0.111	−0.210	− 0.332*	−0.056
TLG	…	…	…	0.839**	−0.187	−0.239	− 0.126	−0.226*	− 0.285*	−0.056
MTV	…	…	…	…	−0.239*	−0.320*	− 0.132	−0.243*	− 0.175	−0.020
ADC_mean_	…	…	…	…	…	0.633**	0.807**	0.471**	0.585**	0.089
ADC_min_	…	…	…	…	…	…	0.564**	0.853**	0.345**	0.039
D_mean_	…	…	…	…	…	…	…	0.683**	0.464**	0.037
D_min_	…	…	…	…	…	…	…	…	0.235*	0.042
f	…	…	…	…	…	…	…	…	…	0.207*
D*	…	…	…	…	…	…	…	…	…	…

* *p* < 0.05

** *p* < 0.01

### The relationship between lymph node metastasis and imaging parameters in patients with PET negative lymph nodes

A total of 76 patients were evaluated: 69 without lymph node metastasis and 7 with lymph node metastasis. SUV_max_ (U test, *p* = 0.016; AUC 0.797, *p* < 0.001), SUV_mean_ (U test, *p* = 0.018; AUC 0.792, p < 0.001), MTV (U test, *p* = 0.013; AUC 0.806, *p* = 0.024), TLG (U test, *p* = 0.004; AUC 0.855, p < 0.001), and D_min_ (U test, *p* = 0.037; AUC 0.758, *p* = 0.005) showed significant between-group differences. The remaining parameters did not show statistical differences between the two groups. The TLG had the strongest predictive value according to the univariate and multivariate logistic analysis in the subset of patients with PET negative lymph nodes (Table 6).

**Table 6:** For patients with PET negative lymph nodes, univariate and multivariate analysis of parameters to predict lympha node metastasis

Method: Stepwise; Dependent Y: Lymphatic metastasis Multivariate logistic regression: Enter Variable< 0.0167				
Univariate logistic regression analysis				
Variable	coeffcients	Std Error	Wald	P
MTV	0.13821	0.047032	8.6359	0.003
TLG	0.0084360	0.0029554	8.1477	0.004
D_min_	−7.32445	3.40917	4.6159	0.032
Multivariate logistic regression analysis				
Variable	coeffcients	Std Error	Wald	P
TLG	0.014966	0.0056818	6.9379	0.0084

## Discussion

Recognition of lympha node metastasis before treatment in patients with cervical cancer is essential for personalized treatment plans, and diagnostic imaging is most commonly used for this purpose. In the present study, all data were measured by integrated PET-MRI. With the integrated device, a shortened scanning time, a reduced radiation dose, and the simultaneous PET and MRI parameters can be achieved, enabling combined multiparameter prediction and correlation [16–19].

The IVIM sequences was introduced into the present PET/MR study, which is an extension of diffusion-weighted imaging (DWI) sequence, to PET/MRI. DWI reflects the diffusion dynamics of tissue water molecules by ADC; however, the attenuation of DWI signals in tissues is determined by both water molecule diffusion and microcirculation perfusion. ADC is a quantitative parameter based on single index model of DWI, and it reflects both the true diffusion of water molecules and the “false diffusion”, caused by the blood microcirculation in the capillaries. This inability to reflect the movement of water molecules in living tissues limits the utility of DWI to evaluate microstructure changes.

The IVIM model compensates for these technical defects of traditional DWI. The theoretical basis is to double-exponentially fit tissue signals based on multiple b-values, and quantitatively extract the main signal attenuation components from the low b-values of the curve. The true diffusion parameter value of the tissue was calculated at high b-values [20]. So, this model has a higher accuracy to reflect the signal attenuation of water molecules than the DWI single exponential model. IVIM models can calculate three parameters, including D (purediffusion coefficient), f (perfusion fraction), and D* (pseudo-diffusion coefficient). In the IVIM model, D and f value are indicators to reflect the true diffusion coefficient and perfusion information of the molecule, respectively. IVIM modalities have also been used in other cervical cancer studies [21, 22]. However, the current IVIM technology still has shortcomings and has not been applied on a large scale in the clinic setting. The limitations include: (1) The organs in the abdominal cavity may have a slight displacement movement during long-time scans, which may affect image post-processing; (2) image distortion caused by magnetic susceptibility artifacts at the gas-soft tissue interface leads to artifacts obscuring lesions or unclear display of lesions, affecting data measurement and (3) imprecise ROIs including normal tissues result in poor measurement repeatability.

We established a combined prediction model for cervical cancer patients with lymphatic metastasis by multiple logistic regression analysis, using TLG and D_min_ of the tumor and PET for lymph node diagnosis while filtering out the remaining parameters. Several mechanisms may explain the strong predictive potential of these 3 combined parameters. In previous studies, lymph node with FDG uptake more than the background lymph node metabolism level was a diagnostic criterion for lymphatic metastasis [8, 9]. Our study confirms the ability to diagnose lymphatic metastasis with this method (AUC 0.736, *p* < 0.001), but also demonstrates its low sensitivity (58.82). In contrast, the combination of tumor TLG and D_min_ and this diagnostic criterion had better predictive value than the diagnostic criterion alone (AUC 0.913, sensitivity 94.12, specificity 82.05, *p* < 0.001; difference between areas 0.176, Z = 3.141, *p* = 0.002). Previous studies have shown that the expression of glucose transporter-1 (Glut-1) is related to lymph node metastasis in a variety of tumors [23, 24], which will trigger an increase in FDG uptake and the corresponding quantitative indicators [25]. TLG is a comprehensive parameter that reflects the metabolic activity of the whole tumor. Compared with SUV_max_, TLG can assess tumor burden more accurately. A study involving patients with early cervical cancer showed that tumor MTV and TLG values are important imaging indicators for predicting lymphatic metastasis [26]. Similar conclusions have been obtained in studies involving endometrial carcinoma patients and thyroid carcinoma patients [27, 28].

Recently, many studies have quantitatively analyzed lymph nodes by IVIM to predict pelvic lymph node metastasis [29, 30]. However, the slice of IVIM imaging is thick, which makes it difficult to carry out clinically concerning the usually small volume of lymph nodes. In this study, we evaluated lymphatic metastasis using IVIM parameters of tumors for cervical cancer such as the D value. Theoretically, the D value is not affected by the microcirculation blood flow of the tumor tissue, giving a more accurate signal on the diffusion of the tissue water molecules than does the ADC value. Studies have suggested that D values correlate with hypoxia and the tumor-stroma ratios [18, 31]. D_min_ value corresponds to the lowest tumor diffusion region, which may more accurately reflect the number and heterogeneity of tumor cells, as well as tumors with lymphatic metastasis.

A study using similar methods suggests that a combination of serum squamous cell carcinoma antigen (SCC-Ag) level, SUV_max_, and lymph node status is an important prognostic indicator for cervical cancer [32]. Another study suggested that the combination of lymph node CT indicators and tumor TLG values can improve the accuracy of PET/CT for the diagnosis of lymphatic metastasis in patients with cervical cancer [33]. Similar combined methods are also applied to breast cancer [34].

A previous study has shown that PET/MR for lymphatic metastasis was mainly dependent on the sensitivity of PET [35]. In this study, PET was also significantly better than MR in the diagnosis of lymph nodes. But we found a large number of PET false-negative cases, which accords with the findings of endometrial cancer and non-small cell lung cancer studies [36, 37]. Identifying PET false-negative cases has always been a clinical challenge. Therefore, we further explored the relationship between lymph node metastasis and imaging parameters in patients with PET/MRI-negative lymph nodes. By multivariate logistic analysis, TLG had the strongest predictive value for lymph node metastasis in this subset of patients.

This study has several limitations. On one hand, our scan range was from the vaginal level to the upper edge of the humerus. Therefore, we could not measure the SUV_peak_ value which may better reflect the aggressiveness of the tumor [25]. On the other hand, we used the pathologic diagnosis as the gold standard. The inclusion of patients who have undergone surgery results in fewer negative cases in the sampled population. A further evaluation of more cases is needed to verify our conclusions.

## Conclusions

In all patients, the combination of tumor TLG and D_min_ of the tumor and PET for lymph node diagnosis had better predictive performance than the other imaging parameters for nodal metastasis. In patients with PET negative lymph nodes, TLG showed the strongest predictive potential. These findings may have clinical applications in personalized treatment planning for patients with cervical cancer.

## Data Availability

The datasets used and/or analyzed during the current study are available from the corresponding author on reasonable request.

## Abbreviations

ADC: Apparent diffusion coefficient
CT: Computed tomography
D: Diffusion-related coefficient
D*: Perfusion related diffusion coefficient
DWI: Diffusion-weighted imaging
F: Perfusion-related parameter
FDG: Fluoro-D-glucose
FIGO: Federation International of Gynecology and Obstetrics
Glut-1: Glucose transporter-1
HE: Hematoxylin-eosin
IVIM: Intravoxel incoherent motion
MRI: Magnetic resonance imaging
MTV: Metabolic tumor volume
PET: Positron emission tomography
ROC: Receiver operating characteristic
ROI: Region of interest
SCC-Ag: Serum squamous cell carcinoma antigen
SUV_max_: Maximum standardized uptake values
SUV_mean_: Mean standardized uptake values
TLG: Total lesion glycolysis
